# Silent Carriers: The Hidden Threat of Antibiotic-Resistant *Staphylococcus aureus* in Retail Seafood Across Poland’s Tri-City Area

**DOI:** 10.3390/antibiotics14010073

**Published:** 2025-01-12

**Authors:** Anita Kukułowicz, Izabela Steinka, Aleksandra Szelągowska

**Affiliations:** Department of Quality Management, Faculty of Management and Quality Sciences, Gdynia Maritime University, 81–87 Morska St., 81-225 Gdynia, Poland; i.steinka@wznj.umg.edu.pl (I.S.); aleksandra.szelagowska@vp.pl (A.S.)

**Keywords:** *Staphylococcus aureus*, antibiotic resistance, antimicrobial susceptibility, seafood, public health

## Abstract

**Background/objectives**: Antibiotic-resistant *Staphylococcus aureus* poses a significant risk to food safety and public health, particularly through the consumption of contaminated seafood. This study aimed to assess the presence and antibiotic resistance of *S. aureus* in seafood sold in the Tri-City area of Poland, addressing a knowledge gap regarding the region. **Methods**: Seafood samples (*n* = 89) were categorized according to their origin: domestic—Poland (PL), European countries (ECs), and Asian countries (ACs). *S. aureus* was isolated using ISO 6888-1 methods, and antimicrobial susceptibility testing was conducted against three antibiotics: erythromycin, clindamycin, and gentamicin, following CLSI guidelines. **Results**: Of the 89 samples, 68.5% were contaminated with *S. aureus*. The highest resistance rates were found for erythromycin (30.5%), with gentamicin showing the lowest resistance (8.3%).No significant correlation (*p* > 0.05) was found between resistance patterns and fish origin or processing level. **Conclusions**: The results of this study highlight the widespread occurrence of *Staphylococcus aureus* in fish sold in the Tri-City area, with a notable prevalence of antibiotic-resistant strains.

## 1. Introduction

Seafood are rich sources of essential nutrients, providing high-quality proteins, healthy fats, and important vitamins like D3 and B12. They are also excellent sources of minerals such as calcium, phosphorus, iodine, selenium, magnesium, and iron. Additionally, fish is a key source of n-3 LC PUFA, taurine, and choline, which contribute to overall health. These nutrients make fish and seafood a valuable part of the diet, especially when other sources of these nutrients are limited [1,2,3,4]. In Poland, seafood consumption totals around 14 kg per person [5]. Projections estimate that by 2031, global seafood consumption will rise to 21.4 kg per person, representing an increase of roughly 1 kg compared to the 2019–2021 average [6].

Seafood can harbor pathogenic bacteria that are harmful to humans, such as *Listeria monocytogenes*, *Salmonella* spp., *Staphylococcus aureus*, and *Escherichia coli*, posing potential risks to consumers. Most foodborne outbreaks are linked to the consumption of raw or inadequately cooked fish [4,7,8]. Between 2001 and 2021, 16 outbreaks caused by *S. aureus* were confirmed, linked to fish, salmon, shrimp, lobster, oysters, sushi, codfish, crab, seafood pasta, and mackerel (both raw and cooked) [4]. *S. aureus* is a non-spore-forming, facultatively anaerobic bacterium that can grow in a wide temperature range (7 °C to 48.5 °C, optimal at 30–37 °C) and pH range (4.2 to 9.3, optimal at 7–7.5). It is known to adapt and survive in low temperatures and high sodium chloride (NaCl) concentrations. Resistant to high osmolarity, detergents, and alcohol due to its cross-linked peptidoglycan, *S. aureus* is easily killed by pasteurization or cooking [9,10]. Additionally, it possesses various virulence factors and toxins that can lead to diseases such as toxic shock syndrome and foodborne infections [11]. While *S. aureus* is not a natural part of the native flora of fish and aquatic products, it can still be isolated from freshly caught fish, especially those from warm waters [12]. Seafood can become contaminated by food handlers who carry *S. aureus* on their skin or in their mucous membranes, as well as from the environment, including soi and water [4,13,14]. The increasing detection of *S. aureus*, especially methicillin-resistant strains (MRSA), in food-producing animals like fish, both pre- and post-harvest, highlights the importance of focusing on this bacterium within the fish food chain [8]. Additionally, the widespread use of antibiotics in animal production and aquaculture—to promote growth, increase feed efficiency, and prevent infections—can contribute to antibiotic resistance in foodborne pathogens, further exacerbating these concerns [15,16]. The problem of antibiotic resistance is steadily escalating. A study conducted in 2019 indicated that 495,000 people worldwide died from infections caused by antibiotic-resistant bacteria. Among these, 1,270,000 deaths were attributed to bacterial pathogens resistant to all available antibiotics. Projections indicate that by 2050, annual mortality rates may reach 10 million people. Deaths attributed to antibiotic resistance are expected to surpass those caused by cancer [17,18].

Rzepkowska et al. [19] and Solomon et al. [20] report that organisms resistant to multiple drugs are often isolated from aquatic food sources, as well as from animal feed and their waste. The incidence of *S. aureus* varies across fish species, but its presence raises significant concerns about the safety and quality of products for human consumption, particularly when the microorganisms are opportunistic or pathogenic [13]. Multidrug resistance (MDR) in *S. aureus* has become a significant concern in foodborne pathogens. Analysis of antibiotic resistance in pathogens isolated from various food products shows that the occurrence rate is generally ≥11%, with β-lactam antibiotics being the most common target of resistance. Furthermore, MDR is observed in at least 36% of these pathogens. Due to their ability to survive for extended periods outside a host—sometimes up to several months—multidrug-resistant *S. aureus* strains represent a growing issue, not only in healthcare settings, but also within the food production industry [21]. This persistence, combined with mechanisms such as the accumulation of diverse resistance genes on plasmids and the action of multidrug efflux pumps, underscores the urgent need for effective control strategies to mitigate the spread of MDR bacteria in the food supply chain [22]. Antibiotic resistance (ABR) is transferred between microorganisms through various mechanisms, with environmental factors playing a crucial role. The food supply chain provides multiple opportunities for the dissemination of ABR genes, allowing antimicrobial-resistant bacteria and resistance genes to be transmitted between humans, animals, and the environment. This interconnected pathway not only raises concerns about food safety, but also poses a potential public health risk [23,24]. Data on the presence of antibiotic-resistant *S. aureus* seafood available for sale in the markets of the Tri-City area of Poland are scarce. The aim of this study was to evaluate the presence of antibiotic-resistant *S. aureus* in fish obtained from retail points in the Tri-City area of Poland.

## 2. Results

### 2.1. Sample Contamination by *S. aureus*

The occurrence rate and the mean counts of *S. aureus* in the analyzed seafood samples, grouped by origin and level of processing, are shown in Table 1. The analysis revealed that the overall occurrence rate of *S. aureus* was 68.5% (61/89), with the highest occurrence rates recorded equally for PL, FS, F/PL, FS/PL and FS/ECs, at 71.4%, while the lowest was observed in F/ECs, at 58.8%. The counts of *S. aureus* in the tested seafood samples ranged from undetected to 3.1 × 10^2^ CFU/g. Considering the origin of the samples, the lowest mean values were obtained for PL, followed by samples from ACs. Although no statistically significant difference was found for the numbers of *S. aureus* between samples of different origins(H(2, *n* = 61) = 2.2058, *p* = 0.3319), higher values were observed in products from ECs (Table 1). Statistical analysis also failed to confirm a dependence of the bacterial count on the degree of processing (H(1, *n* = 61) = 0.2277, *p* = 0.6333). The highest bacterial count of *S. aureus* contamination in fresh products was observed in salmon (3.1 × 10^2^ CFU/g) originating from Poland, whereas in frozen products, the highest count (2.4 × 10^2^ CFU/g) was recorded in pangasius sourced from Vietnam.

### 2.2. Antimicrobial Susceptibility Characterization

Among the 82 analyzed samples, 43.9% (*n* = 36) showed growth of *S. aureus*, which allowed the preparation of an appropriate suspension (with a density of 0.5 on the McFarland scale), and antibiotic resistance was assessed for these isolated strains. Based on the inhibition zone values around the antibiotic disks, the tested strains were classified as resistant or sensitive. Table 2 presents the percentages of strains classified as resistant and multidrug-resistant (MDR)to the tested antibiotics. Among the analyzed *S. aureus* strains, 11 were resistant to erythromycin, while only 3 were resistant to gentamicin.

No multidrug-resistant (MDR) strains were identified among the tested isolates. Three strains, isolated from frozen salmon (ECs), frozen carp (ACs), and fresh salmon (PL), were resistant to erythromycin and clindamycin. Two strains, isolated from frozen mussels (ECs) and fresh cod (ECs), were resistant to clindamycin and gentamicin, while a strain isolated from fresh carp (PL) was resistant to erythromycin and gentamicin. A total of 21 strains (14 from frozen products and 7 from fresh products) were susceptible to all tested antibiotics. After comparing all the tested samples based on their origin and level of processing, it was found that the occurrence of *S. aureus* strains resistant to the tested antibiotics was not dependent on these factors. The analyzed relationships were not statistically significant (*p* > 0.05).

## 3. Discussion

The presence of *S. aureus* strains in fishery products is not a natural occurrence. However, factors such as inadequate hygiene and sanitary conditions during the processing and transportation of fish and seafood, cross-contamination during storage, and contamination by asymptomatic carriers of *S. aureus* among workers all contribute to the introduction of this bacterium into seafood [25,26]. The frequency of occurrence of *S. aureus* in the analyzed seafood samples was 68.5% (61/89). Literature reports indicate varying rates of *S. aureus* occurrence in fishery products. Kumar et al. [26] identified *S. aureus* in 10.3% of fishery products. Abrahim et al. [27] observed a significantly higher prevalence, with 24% of rainbow trout samples testing positive for *S. aureus*, while Dallal et al. [28] detected the bacterium in 28% of both frozen and fresh shrimp samples. Higher prevalence rates were recorded by Obaidat [14] in fresh fish (47.3%), Fri et al. [29] in aquaculture fish (62.9%), Hammad et al. [30] in ready-to-eat fish (87%), and Anjusha et al. [31], who reported a rate as high as 90%. The increased prevalence of *S. aureus* in these products may be attributed to poor hygienic practices among fish handlers and contact with contaminated surfaces [14,32]. Although no statistically significant difference was observed between the number of *S. aureus* and the degree of processing of the samples (H(1, *n* = 61) = 0.2277, *p* = 0.6333), a higher prevalence was found in FS products compared to F ones (Table 1). Sivaraman et al. [33] reported a twofold higher incidence of *S. aureus* in fresh fish (18.5%) compared to frozen fish (9.4%). However, Dallal et al. [28] found a lower incidence of these bacteria in fresh products (22%) than in frozen products (34%). Cooling and freezing can enhance the safety and extend the shelf life of food products; however, *Staphylococci* tend to be relatively resistant to the adverse effects of freezing [15]. FS products were found to be contaminated with *S. aureus* at an average count of 1.2 × 10^2^ CFU/g. Pyz-Łukasik and Paszkiewicz [7] did not detect the presence of these bacteria in the fish samples they studied, while Samy et al. [34] reported an average count of *S. aureus* at 1.82 × 10^7^ CFU/g. In the analyzed F products, the average count of *S. aureus* was 1.2 × 10^2^ CFU/g. Edris et al. [35] found a slightly lower count of these bacteria in frozen fish, at 2.8 × 10^1^ CFU/g, while Samy et al. [34] reported a significantly higher count, at 7.3 × 10⁶ CFU/g. The bacteriological quality of frozen products depends on the bacterial content of the raw material, contamination during handling and processing, and the extent to which these contaminants are removed [36]. Processing technologies, such as freezing, can reduce the bacterial load of foods [37], as observed in this study (Table 1). Low temperatures typically inhibit or stop microbial growth, but often do not kill bacteria. While freezing can kill some microorganisms through physical damage, others may be sublethally injured, and can recover, potentially becoming infectious [36,38]. The European Commission Regulation 2073/2005 [39] establishes process hygiene criteria, setting limits of 10^2^ to 10^3^ CFU/g for coagulase-positive *Staphylococci* in shelled and shucked products of cooked crustaceans and molluscan shellfish. If levels exceed 10^3^ CFU/g, corrective measures to improve production hygiene are required. Although none of the tested seafood samples exceeded the 10^3^ CFU/g threshold, 40.4% (36/89) surpassed the lower limit of 10^2^ CFU/g, highlighting the importance of ongoing surveillance and the potential necessity for enhancing hygiene practices to prevent further bacterial growth.

Although this study focuses on the Tri-City area of Poland, its findings contribute valuable baseline data on the prevalence of antibiotic-resistant *S. aureus* in retail seafood, which is currently under-represented in Polish research. These results not only highlight the need for ongoing monitoring in this region, but also serve as a basis for future studies comparing resistance patterns in other regions of Poland and Europe. In analyzing the resistance mechanisms of *S. aureus* isolates in seafood, certain genetic determinants associated with antibiotic resistance provide context for our phenotypic results. Previous studies have identified key genes responsible for resistance to common antibiotics, which may also be found in *S. aureus* isolates from seafood. For instance, high resistance to erythromycin may be associated with the presence of erm genes (ermA, ermB, and ermC), which encode rRNA N-6-adenine methyltransferase enzymes that modify the ribosome, leading to resistance to macrolides, lincosamides, and streptogramins B (collectively known as MLS antibiotics). Similarly, vancomycin resistance could involve the vanHAXYZ gene cluster, conferring resistance through glycopeptide modification via transposons like Tn1546 [40,41]. The presence of these genetic determinants in foodborne *S. aureus* isolates has significant implications for public health and the spread of resistance. Genes such as mecA, which encodes the PBP2a protein responsible for methicillin resistance, are embedded in mobile genetic elements (e.g., SCCmec) that facilitate their horizontal transfer, potentially spreading resistance across various *S. aureus* strains and complicating treatment protocols [41,42].Additionally, determinants such as *aacA-aphD* for aminoglycoside resistance, which is often regulated by beta-lactam antibiotics, and *blaZ* for β-lactam resistance, underscore the role of mobile genetic elements like plasmids and transposons in enhancing resistance mechanisms [40,43]. These genetic factors contribute to the persistence and spread of antibiotic-resistant strains, particularly when hygiene practices and environmental controls in food handling and processing environments are inadequate, facilitating the transfer and proliferation of these resistant bacteria. *S. aureus* is notable for its virulence and multidrug resistance, which it can acquire through mechanisms like spontaneous mutations, transduction, conjugation, and transformation [44]. Antibiotic-resistant *S. aureus* can be found in raw foods of both animal and plant origin, posing a potential risk to food safety and public health [45]. The global trade of fish increases the risk of cross-continental transmission of multidrug-resistant and enterotoxigenic *S. aureus*, necessitating close monitoring due to its potential impact on consumer health worldwide [32]. While molecular testing in this study was not conducted to confirm these specific genes, future research integrating genetic analysis would elucidate the actual presence and diversity of these determinants, allowing for a clearer understanding of the spread and persistence of antibiotic-resistant *S. aureus* in the seafood industry. Obaidat et al. [14] demonstrated that out of 156 *S. aureus* isolates, 138 (88.5%) were resistant to at least one antibiotic. In our own study, this percentage was lower, at 25% (9/36). Abrahim et al. [27] demonstrated that all the *S. aureus* isolates they obtained from fish were multidrug-resistant, exhibiting resistance to 2–13 antibiotics. In our study, isolates resistant to 2–3 antibiotics accounted for 16.7% of the total isolates. Resistance to at least two antibiotics was observed in 16.7% of the isolates, which is lower than the 35.4% reported by Obaidat et al. Additionally, no isolates in our study were resistant to at least three antibiotics, a finding significantly lower than the 30.5% prevalence reported by Obaidat et al. In this study, the lowest resistance rates were observed for gentamicin (8.7%) and clindamycin (22.2%), while erythromycin exhibited the highest resistance rate, at 30.5%. In contrast, Obaidat et al. [14] reported low resistance rates for erythromycin, tetracycline, and clindamycin. Rashid et al. [46] found resistance rates of 38% for gentamicin. Onmaz et al. [47] demonstrated that all *S. aureus* isolates were susceptible to gentamicin, which differs from our findings. Their erythromycin resistance rates were similar, at 33%. Additionally, Phan et al. [48] demonstrated that *S. aureus* isolates from *Pangasius* fish were resistant to gentamicin (44%) and erythromycin (32%), further highlighting variability in resistance profiles depending on the source of isolation. Similarly to the findings of Obaidat et al. [14], no statistically significant differences (*p* > 0.05) were observed between samples of different countries of origin in terms of antibiotic resistance.

Rashid et al. [46] demonstrated that antibiotic resistance, combined with biofilm formation, in *S. aureus* isolates, including those from fish, poses a significant challenge. Biofilm production, a key virulence factor, enhances bacterial pathogenicity and increases infection risk. Together, these traits—antibiotic resistance and biofilm formation—substantially elevate the risk of foodborne infections. Moreover, animal-derived food products provide an ideal environment for the development and persistence of *S. aureus* biofilms, acting as both a surface for adhesion and a reservoir for bacterial growth [49].

### Network Analysis of Key Concepts

**Figure 1:**
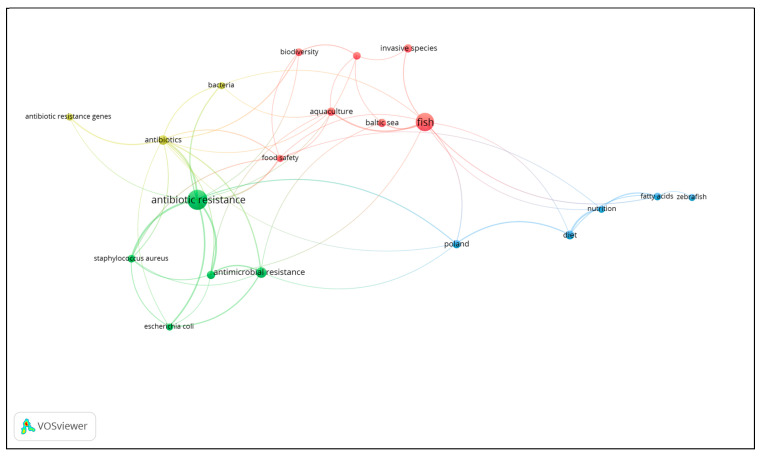
Network visualization of the links between the keywords “fish”, “seafood”, “*S. aureus*”, “antibiotic resistance”, and “Poland”, among others.

The network visualization generated using VOSviewer 1.6.20 (Figure 1) illustrates the relationships between key concepts related to the topic of this study, such as “fish”, “seafood”, “*S. aureus*”, “antibiotic resistance”, and “Poland”. The visualization was optimized by limiting the number of analyzed keywords and applying filters covering the period of 2021–2024 and the country “Poland”.

The map consists of 20 items (four clusters), each marked with a distinct color. Each cluster groups conceptually related terms, indicating thematic similarity and the strength of interconnections between them. The map displays five key nodes, which serve as central elements of the analysis. The visualization shows that some concepts are more central than others, which is evident in the size of the nodes. Larger nodes represent concepts that are more frequently linked to other terms and have stronger interconnections. The largest nodes on the map are “antibiotic resistance”, “fish”, “*S. aureus*”, “Poland”, and “diet”. “Antibiotic resistance” serves as a central node connecting several clusters, highlighting the interdisciplinary nature of the issue, which links aspects of food safety, public health, and pathogens. “Fish” is a key node related to seafood production and its nutritional value. This node is connected to concepts such as “diet”, “aquaculture”, and “Baltic Sea”, which indicates the link between fish production and consumption in both regional and global contexts. The “*S. aureus*” node is particularly important in the context of food safety and foodborne pathogens. This concept is directly linked to “antibiotic resistance” and “bacteria”, which underscores the critical role of *S. aureus* as a risk factor in fish products. “Poland” is connected to the main research themes, highlighting the role of the regional context within the broader global context of research on antibiotic resistance. The last of the main nodes, “diet”, is linked to concepts such as “fish”, “nutrition”, and “fatty acids”, which underscores the role of fish and seafood as valuable components of the human diet. Direct connections on the map indicate that “Poland” is directly linked to the following concepts: “fish”, “antibiotics”, “antibiotic resistance”, “antimicrobial resistance”, and “diet”. These connections highlight the importance of research on the Polish seafood market, especially in the context of food safety and antibiotic resistance. Indirect connections link “Poland” to concepts such as “food safety”, “Baltic Sea”, “aquaculture”, “bacteria”, and “*S. aureus*”. This demonstrates the close relationship between the Polish seafood market and global challenges related to food safety and foodborne pathogens. This visualization highlights the interconnections between research themes related to antimicrobial resistance in seafood. In particular, it emphasizes the role of “antibiotic resistance” as a key node linking different research areas, including fish production, aquaculture, and pathogen analysis. The analysis of interactions between key concepts also reveals the critical role of “*S. aureus*”, which was found in 68.5% of the analyzed seafood samples. This underscores the importance of this pathogen in the context of food safety. “Fish” is also a significant node, reflecting the importance of fish and seafood as both carriers of antibiotic-resistant bacteria and as a source of essential nutrients. Moreover, “food safety”, as an element indirectly connected to “Poland”, “fish”, and “*S. aureus*”, indicates that this study is significant not only in the local context, but also globally, since research on antibiotic resistance and pathogens in fish is crucial for international food safety. This visualization shows that key research concepts, such as “antibiotic resistance”, “fish”, “*S. aureus*”, and “food safety”, are closely interconnected. Each of these elements represents an integral part of research on antibiotic resistance within the food supply chain. The relationship between them is visible on the map, where thicker lines connect nodes with stronger links, indicating their more frequent co-occurrence in the scientific literature. In particular, the strong connection between “antibiotic resistance” and “*S. aureus*” emphasizes the relationship between antibiotic resistance and the presence of this pathogen in fish and seafood. Similarly, the direct connection between “Poland” and the concepts “antibiotic resistance”, “food safety”, and “*S. aureus*” confirms the significance of local studies as part of a broader discussion on the global problem of food safety. In summary, the map confirms that research conducted in Poland is relevant to the global discussion on antibiotic resistance and food safety. Furthermore, these findings indicate that the Polish seafood market has a direct impact on food safety, which may serve as a basis for future comparative studies at the international level.

## 4. Materials and Methods

### 4.1. Study Area

The Tri-City metropolitan area in northern Poland comprises three cities: Gdańsk, Gdynia, and Sopot (coordinates: 18°83′40″ E, 54°82′70″ N). These cities are aligned along the coast of the Gdańsk Bay in the Baltic Sea. The bay’s western section is characterized by the shallow waters of the Bay of Puck, while the southeastern part features the Vistula Lagoon, separated from the open sea by the Vistula Spit and connected through the Strait of Baltiysk. The Tri-City area was selected as the research area due to its reputation as one of Poland’s most popular tourist destinations and its long-standing connection to fisheries and the consumption of aquatic products, which are deeply rooted in the local culture. Tourists visiting the region frequently opt for fresh fish for lunch, highlighting the importance of local culinary traditions based on seafood.

### 4.2. Materials

The tests were conducted on seafood (including fish, crustaceans, and mollusks) purchased from various sales points across the Tri-City area (*n* = 89, Table 3), according to their current availability. The products were divided into three categories: seafood caught in the Baltic Sea region and sourced from domestic farms (PL), seafood entering the Polish market from countries within Europe (ECs), and seafood imported from Asian and South American countries (including Bangladesh, India, Argentina, Chile, Vietnam, and China) (ACs).

Data on the presence of antibiotic-resistant *S. aureus* in seafood available for sale in the markets of the Tri-City area of Poland are scarce. The aim of this study was to evaluate the presence of antibiotic-resistant *S. aureus* in seafood obtained from retail points in the Tri-City area of Poland.

### 4.3. Isolation of Staphylococcus aureus

All the samples were analyzed for the enumeration and isolation of total *Staphylococci* and *S. aureus* using the method outlined by the International Organization for Standardization (ISO 6888-1). Enumeration and isolation were performed on Baird-Parker + RPF agar medium (bioMérieux, Poland, Warsaw). For each sample, a 10 g portion of the product to betested was collected under sterile conditions and homogenized with 90 mL of buffered peptone water, resulting in a 1:10 dilution of the initial sample. Further decimal dilutions (e.g., 1:100) were prepared as necessary. A 1 mL aliquot from each dilution was plated using the pour plate technique. Plates were incubated at 37 °C for 24–48 h. Typical black colonies with opaque halos on BP+RPF agar were considered presumptive of *S. aureus*, and its presence was confirmed through catalase and coagulase testing [13,15,50].

### 4.4. AntimicrobialSusceptibility Testing

Antimicrobial susceptibility testing was performed on all the obtained microbiological isolates (*n* = 36) using three antimicrobial agents (BioMaxima S.A, Gdansk, Poland): erythromycin (15 μg), clindamycin (2 μg), and gentamicin (10 μg). The Kirby–Bauer disk diffusion method was employed, following the Clinical and Laboratory Standards Institute (CLSI) guidelines [51]. Each *S. aureus* isolate was inoculated onto Mueller–Hinton II agar plates (BioMaxima S.A, Gdansk, Poland), which were impregnated with antibiotic disks of varying concentrations, as outlined above, at the 0.5 McFarland standard. The plates were incubated at 37 °C for 18–24 h, after which the zones of inhibition surrounding each disk were measured according to CLSI criteria, and the isolates were categorized as sensitive, resistant, or intermediate. Multidrug resistance (MDR) was defined as the resistance of a given isolate to at least three of the tested antibiotics. To determine MDR, the results of each isolates were analyzed individually. If an isolate exhibited resistance to three or more antibiotics, it was classified as MDR [52,53]. The MDR rate (%) was calculated as the percentage of isolates showing resistance to three or more antibiotics out of all the tested strains (*n* = 36).

### 4.5. Statistical Analysis

The normality of the samples was assessed using the Shapiro–Wilk test. The relationship between the occurrence of *S. aureus* in the samples and the method of processing, as well as the origin of the products, was determined using Kruskal–Wallis rank ANOVA. To evaluate the association between qualitative variables and the presence of antibiotic-resistant *S. aureus* in the samples, the chi-square test was applied. The significance level was set at *p* < 0.05. Data analysis was performed using Statistica 13 software (StatSoft, Inc. Palo Alto, CA, USA) [54].

## 5. Conclusions

The findings of this study highlight the widespread occurrence of *Staphylococcus aureus* in fish sold in the Tri-City area, with a notable prevalence of antibiotic-resistant strains. The highest resistance rates were observed for erythromycin, while gentamicin exhibited the lowest resistance. Notably, no multidrug-resistant (MDR) isolates were identified in this study, suggesting a relatively low current risk from highly resistant strains in this region. However, the presence of antibiotic-resistant *S. aureus* emphasizes the ongoing need for continuous monitoring of antimicrobial resistance in seafood products. No significant relationships were found between the origin or level of processing of the seafood and the occurrence of antibiotic-resistant strains, underscoring the complexity of resistance development. These results contribute valuable baseline data for understanding resistance patterns in Polish seafood, and emphasize the importance of global surveillance to address the risks associated with antimicrobial resistance in the seafood supply chain. Future studies should expand the geographic scope, increase sample sizes, and explore additional factors such as handling practices, processing environments, and cross-contamination during distribution. Incorporating molecular analyses would also provide deeper insights into the genetic mechanisms underlying resistance, and inform strategies to mitigate public health risks.

## Figures and Tables

**Table 1:** Occurrence and count of *S. aureus* in various product samples on Baird-Parker Agar + RPF.

	No. of Samples	M ± SD [CFU/g]	No. of Samples Positivefor *S. aureus* (%)
	**Place of origin**		
PL	35	1.1 × 10^2^ ± 6.6 × 10^1^	25 (71.4)
ECs	31	1.3 × 10^2^ ± 6.4 × 10^1^	20 (64.5)
ACs	23	1.2 × 10^2^ ± 6.9 × 10^1^	16 (69.6)
	**Degree of processing**		
F	47	1.2 × 10^2^ ± 4.3 × 10^1^	31 (66.0)
FS	42	1.2 × 10^2^ ± 6.6 × 10^1^	30 (71.4)
	**Origin and processing**		
F/PL	7	1.4 × 10^2^ ± 1.6 × 10^1^	5 (71.4)
F/ECs	17	1.2 × 10^2^ ± 4.7 × 10^1^	10 (58.8)
F/ACs	23	1.2 × 10^2^ ± 6.9 × 10^1^	16 (69.6)
FS/PL	28	9.9 × 10^1^ ± 7.2 × 10^1^	20 (71.4)
FS/ECs	14	1.5 × 10^2^ ± 7.7 × 10^1^	10 (71.4)
FS/ACs	0	-	0
Total	89	1.2 × 10^2^ ± 6.6 × 10^1^	61 (68.5)

**Table 2:** Antibiotic resistance and multidrug resistance (MDR) profiles of *S. aureus* isolates.

Antibiotic (Antibiotic Content in Disk)	Resistance (%)	MDR (%)
Erythromycin (15 μg)	30.5	
Clindamycin (2 μg)	22.2	
Gentamicin (10 μg)	8.3	
Multidrug resistance		0

**Table 3:** Types of tested products.

**Place of Origin**	
PL	*n*= 35
ECs	*n*= 31
ACs	*n*= 23
**Degree of Processing**	
F	*n*= 47
FS	*n*= 42

Number of samples (*n*); frozen—F, fresh/raw—FS.

## Data Availability

The data are contained within the article or Supplementary Materials.

## Supplementary Materials

The following supporting information can be downloaded at: https://www.mdpi.com/article/10.3390/antibiotics14010073/s1.
